# Comparative Transcriptome Analysis of Resistant and Susceptible Common Bean Genotypes in Response to Soybean Cyst Nematode Infection

**DOI:** 10.1371/journal.pone.0159338

**Published:** 2016-07-21

**Authors:** Shalu Jain, Kishore Chittem, Robert Brueggeman, Juan M. Osorno, Jonathan Richards, Berlin D. Nelson

**Affiliations:** 1 Department of Plant Pathology, North Dakota State University, Fargo, North Dakota, 58108, United States of America; 2 Department of Plant Sciences, North Dakota State University, Fargo, North Dakota, 58108, United States of America; University of Guelph, CANADA

## Abstract

Soybean cyst nematode (SCN; *Heterodera glycines* Ichinohe) reproduces on the roots of common bean (*Phaseolus vulgaris* L.) and can cause reductions in plant growth and seed yield. The molecular changes in common bean roots caused by SCN infection are unknown. Identification of genetic factors associated with SCN resistance could help in development of improved bean varieties with high SCN resistance. Gene expression profiling was conducted on common bean roots infected by SCN HG type 0 using next generation RNA sequencing technology. Two pinto bean genotypes, PI533561 and GTS-900, resistant and susceptible to SCN infection, respectively, were used as RNA sources eight days post inoculation. Total reads generated ranged between ~ 3.2 and 5.7 million per library and were mapped to the common bean reference genome. Approximately 70–90% of filtered RNA-seq reads uniquely mapped to the reference genome. In the inoculated roots of resistant genotype PI533561, a total of 353 genes were differentially expressed with 154 up-regulated genes and 199 down-regulated genes when compared to the transcriptome of non- inoculated roots. On the other hand, 990 genes were differentially expressed in SCN-inoculated roots of susceptible genotype GTS-900 with 406 up-regulated and 584 down-regulated genes when compared to non-inoculated roots. Genes encoding nucleotide-binding site leucine-rich repeat resistance (NLR) proteins, WRKY transcription factors, pathogenesis-related (PR) proteins and heat shock proteins involved in diverse biological processes were differentially expressed in both resistant and susceptible genotypes. Overall, suppression of the photosystem was observed in both the responses. Furthermore, RNA-seq results were validated through quantitative real time PCR. This is the first report describing genes/transcripts involved in SCN-common bean interaction and the results will have important implications for further characterization of SCN resistance genes in common bean.

## Introduction

Common bean (*Phaseolus vulgaris* L.) is the most important edible legume crop worldwide with crop value equal to the combined value of all other food legumes such as pea (*Pisum sativum* L.), chickpea (*Cicer arietinum* L.), peanut (*Arachis hypogaea* L.) and lentil (*Lens culinaris* Medik.) [1, 2]. Beans are the primary source of protein (22% of seed weight) for over 4 billion people in developing countries (FAO: http://faostat.fao.org/). Beans in combination with cereals are a complete source of proteins and other micro/macronutrients and can help eliminate malnutrition. North Dakota and northern Minnesota are the top producers of beans in the United Stated with about 50% of the production (http://www.usdrybeans.com/resources/production/production-facts/). Principal market classes grown in this region are pinto, navy, black, and kidney beans.

Soybean cyst nematode (*Heterodera glycines* Ichinohe; SCN) is an obligate sedentary endoparasite and the most destructive pathogen of soybean (*Glycine max* L.) in the world causing losses of approximately $1 billion annually in the USA alone [3]. SCN does not cause unique aboveground symptoms and can be confused with symptoms of nutrient deficiency, drought stress, herbicide injury or soil-borne fungal disease. SCN can spread readily from field to field in soil particles on farm machinery. SCN has been found in every soybean producing state in the United States except New York and West Virginia [4]. In North Dakota and northern Minnesota, SCN was first found in soybean fields in 2003. There are differences in virulence within the SCN population with virulent types termed HG types. In North Dakota and northern Minnesota the predominant virulent type is HG 0, previously known as race 3. The primary management of SCN in soybean is the use of resistant cultivars and crop rotation with non-host crops to reduce nematode populations in the soil [5]. The soybean genotypes Peking and PI88788 are the major sources of SCN resistance in commercial soybean varieties. However, due to shifts in virulent HG types, the major source of SCN resistance from PI88788 used in most commercial soybean cultivars is now less effective in some US production areas since new HG types such as HG 2.5.7 can reproduce on that resistance source [6]. Thus, it is imperative to identify novel resistance genes from different sources to incorporate more effective genes into cultivars for resistance to predominant SCN HG types. Resistance to SCN is quantitative and major QTLs have been mapped on chromosomes 18 (*rhg1*) and 8 (*Rhg4*) in many soybean cultivars [7]. The SCN resistance of *rhg1* is contributed by multi-copy *rhg1* haplotypes forming two distinct resistant groups i.e. high copy number *rhg1* accessions and low copy number *rhg1* accessions [8]. High copy number *rhg1* group (PI 88788-type) contain 7–10 copies of a 31 kb *rhg1* repeat segment while the low copy number *rhg1* group (Peking-type) contains 3 copies of the *rhg1* repeat along with a newly identified allele of Glyma18g02590. Three different genes encoding a predicted amino acid transporter (Glyma18g02580), an α–SNAP protein (Glyma18g02590) and a protein with wound-inducible protein 12 (WI12) (Glyma18g02610) are present in 31 kb *rhg1* repeat segment and contribute to SCN resistance [8]. The SCN resistance of *Rhg4* is controlled by a serine hydroxymethyl transferase gene [9]. *Rhg4* exhibits dominant gene action for resistance to race 3 of SCN [10]http://www.nature.com/nature/journal/v492/n7428/full/nature11651.html-ref5 and is one of the major QTLs controlling soybean resistance to SCN race 3 (HG type 0) in cv. Forrest [11]. Roots of plants carrying *Rhg* genes are penetrated by infective juveniles, but feeding cells ultimately degenerate, causing the nematodes to die before reaching adult stages.

Some common bean genotypes are considered excellent hosts for SCN [12]. Averaged over a selection of genotypes, Poromarto and Nelson [13] reported that kidney beans are highly susceptible, navy and pinto beans are moderately susceptible and black beans are moderately resistant. These results indicated high susceptibility to moderate resistance that could be grouped by market class and/or genetic origin and background. The reduction of plant growth and seed yield in three different bean classes (pinto, kidney and navy) from HG 0 under field conditions indicated a potential threat to the common bean industry [14]. There is serious concern that SCN could become a major problem for common bean production in the North Dakota-Minnesota region. Black bean is one of the major classes in this region and is moderately resistant. However, there are no known commercial bean varieties with high levels of resistance to SCN in the other three major bean classes, i.e. pinto, navy and kidney, grown in this area. This potential threat of SCN to common bean production has initiated breeding efforts at North Dakota State University to incorporate SCN resistance into bean germplasm for eventual production of SCN resistant varieties.

Screening of the USDA core collection of *Phaseolus vulgaris* germplasm against SCN HG type 0 has identified resistant genotypes in some accessions [15]. However, the genetic control of SCN resistance in common bean is completely unknown. Understanding the genetics of resistance will aid in the development of resistant varieties. In addition, the findings from common bean could have applications in soybean as well given the high level of synteny reported between the two species at the genomic level [16].

Common bean (2n = 22) belongs to the Leguminosae (Fabaceae) family. It has a genome size of approximately 521.1 Mbp consisting of few duplicated loci [17] and about 49% transposable elements [18]. With the availability of genome sequence information for common bean [17], transcriptome analyses may be a fast and cost-effective way to find differentially expressed genes under biotic stress, ultimately leading to the characterization of signaling pathways in the SCN defense response. With the advent of next-generation sequencing technologies that provide relatively cheap deep-sequencing, RNA-seq has become the best approach for transcriptome profiling [19]. Comparative transcriptome analysis using RNA-seq has been successfully performed in various non-model and model species [20]. RNA-seq has been successfully used in identifying differentially expressed genes (DEGs) during SCN infection in soybean [21, 22] and cereal cyst nematode infection in *Aegilopus variabilis* [23]. However, similar studies have not been conducted in common bean. Resistance genes to SCN HG types have been identified in soybean and are frequently utilized in breeding programs to develop resistant cultivars [24, 25]. Soybean is a close relative to common bean and extensive macrosyntentic relationships exist between the species [16, 26]. The synteny blocks between these two species can help in identifying the orthologous genes in common bean involved in the SCN resistance mechanism. The availability of the RNA-seq information of common bean will accelerate the gene identification related to SCN resistance

To understand the mechanism of SCN resistance in common bean, it is necessary to characterize differential gene expression as the result of the host parasite interactions which will provide clues to the physiological changes and reprogramming that occur in the host during compatible and incompatible interactions. Such information could lead to the identity of host genes involved in the resistance response and provide valuable clues as to the specific mechanisms involved in resistance. In addition, the identity of those genes with functional polymorphism contributing to incompatible interactions with SCN could improve our ability to identify resistance in common bean germplasm and improve the breeding efforts by identifying molecular markers to incorporate resistance into commercial bean varieties. The objective of this study was to identify differentially expressed genes that are biologically relevant for establishment of incompatibility during SCN infection in common bean and lay the foundation for identification of genes involved in resistance mechanisms. The study also provides a transcriptome framework for comparing and identifying differential physiological changes occurring in resistant and susceptible plants during SCN infection.

## Materials and Methods

### Plants, egg sources and inoculation

Two pinto bean genotypes GTS-900 and PI533561 were used for the analysis in this study. GTS-900 is a pinto bean variety released by GEN-TEC seeds Ltd. with high seed quality and is resistant to race 1 of bean common mosaic virus and anthracnose. Additionally, GTS-900 is susceptible to SCN HG type 0 [13, 15]. Pinto bean plant introduction (PI) line PI533561 was selected from the USDA core collection of *Phaseolus vulgaris* based on resistance to SCN HG 0 and good agronomic qualities such as plant architecture, seed shape and color and overall plant health. The plant growth system and inoculation method were described previously [13]. Following surface disinfection with 1.0% NaOCl and a subsequent water rinse, seeds were germinated on germination paper for 3 days. Healthy germinated seedlings of uniform size were transplanted into individual plastic “Cone-tainers” (Suewe & Sons, type SC10 Super Cell) containing autoclaved river sand, then inoculated with 2000 eggs of SCN HG type 0 in a water suspension. The SCN egg source was originally obtained from an infested soybean field in North Dakota. Control plants were mock-inoculated with water. The Cone-tainers were placed in autoclaved sand in plastic pots, 30.5 cm in diameter by 30.5 cm deep (Cambro, Huntington Beach, CA), and immersed in a water bath at 27±3°C in a greenhouse. Plants received both natural and supplemental light using high-pressure sodium lamps (1,000 μE m^–2^ s^–1^) for 16 h/day. Before conducting the gene expression profiling, the resistance response of the two genotypes was verified by inoculating five plants from each genotype, then determining the female index after 30 days growth at 27±3°C. Female index is a measurement of the ability of a SCN population to reproduce on a genotype and was calculated as described in Poromarto and Nelson [13].

### Total RNA extraction and purification of mRNA from total RNA

Two independent biological replicates per treatment were grown at different times under the same conditions as described above and entire root tissue was collected eight days post inoculation (dpi) with SCN. Roots were washed in tap water to remove sand and then stored in liquid nitrogen until RNA extraction. Treatments were as follow: PI 533561 non-inoculated (RC), PI 533561 SCN inoculated (RI), GTS-900 non-inoculated (SC), and GTS-900 SCN inoculated (SI). Root tissue (100 mg) was frozen in liquid nitrogen and ground to a fine powder with a mortar and pestle. A total of 3 ml of TRIzol (Invitrogen, Carlsbad, CA, USA) was added to the ground tissue and the sample was split in half for column purification with the TRIzol Plus RNA purification kit (Invitrogen, Carlsbad, CA, USA). The additional step of on-column DNA digestion was performed with DNase I (Invitrogen, Carlsbad, CA, USA) to remove contaminating DNA. RNA was eluted in 250 μl of water per spin column. Equal amount of RNA from five individual plants per replicate of each treatment were pooled together for further analysis. Poly-A RNA was isolated from total RNA with the MicroPoly(A)Purist ^™^ kit using the mRNA spin-column protocol (Ambion Inc., Austin, Texas, USA). A second round of oligo (dT) selection was performed and purified mRNA was eluted in a total of 20 μl of RNA storage solution. Total RNA and mRNA quality was assessed with an RNA 6000 Pico kit on an Agilent 2100 Bioanalyzer (Agilent Technologies, Santa Clara, CA, USA).

### Fragmentation, cDNA synthesis, library and template preparation and sequencing

Purified mRNA was used to construct a full length cDNA pool using Ion Total RNA-Seq Kit v2 (Life Technologies, Carlsbad, CA, USA). The diluted cDNA library was sequenced as per manufacturer’s instructions on Ion Torrent PGM^™^ sequencer at North Dakota State University.

### Mapping reads to reference genome

Quality checks for the RNA-seq libraries were performed using FastQC (http://www.bioinformatics.bbsrc.ac.uk/projects/fastqc) and FastX toolkit (http://hannonlab.cshl.edu/fastx_toolkit/). Estimates of over-represented sequences and quality score distribution were obtained from FastQC. FastX_clipper, and Fastx_quality_trimmer programs of FastX tool kit were used to remove adaptor sequences and retain reads that had a minimum length of 36 bp with a minimum quality score of 20. Reads were mapped to the *Phaseolus vulgaris* reference genome assembly (ftp://ftp.jgi-psf.org/pub/compgen/phytozome/v9.0/Pvulgaris/) using TMAP 3.4.1 (http://github.com/iontorrent/tmap).

### Calculation, normalization, and profiling of gene expression

Alignments generated using TMAP were used for transcript abundance estimation and differential gene expression analyses as described in Trapnell et al. [27]. Normalized transcript abundances were estimated as fragments per kilobase per million mapped reads (fpkm) using Cufflinks version 2.1.1 [28]. Differential gene expression analysis was conducted using CuffDiff program of Cufflinks [29] at a false discovery rate (FDR) of 0.05 after Benjamini-Hochberg correction for multiple testing. Genes were considered significantly differentially expressed only when the fold change was at least two (>1 or < -1 in log2 ratio value), and *P* < 0.05. Venn diagrams were prepared using VENNY [30] to compare resistant and susceptible DEGs.

### Gene Ontology and enrichment analyses

DEGs were mapped to Gene Ontology (GO) database using Blast2GO (http://www.blast2go.de) [31]. For the GO functional enrichment procedure, the total gene set was considered as a background list and DEGs as the screened list obtained from the background list to calculate p-values using hyper-geometric test (http://geneontology.org/page/go-enrichment-analysis). A functional category was considered significantly enriched only when the p*-* value for that category was < 0.05. Significantly enriched DEGs were assigned to categories of biological processes since they represent an overall biological objective that a gene or gene product contributes to a cellular response to a biotic stress [32].

### Validation of RNA-seq data by quantitative real time PCR (qPCR)

Based on potential functional importance, 10 genes were selected for validation by qPCR. Primers were designed using Primer-Blast [33] using default parameters from exon sequences covering introns. qPCR was performed using two biological replicates in two steps. First, RNA was reverse transcribed using QuantiTech^R^ reverse transcription kit (Qiagen, Valencia, CA, USA), which includes genomic DNA elimination as well as reverse transcription. The second step was performed using iTaq Universal SYBR green supermix (BioRad, Hercules, CA) in a BioRad iQ^™^ 5 real-time PCR system following these cycle conditions: 95°C for 30 sec, followed by 40 cycles of 95°C for 10 sec, and 30 s at 60°C. Relative expression levels were calculated using the 2^–ΔΔCt^ method for each gene and were normalized to the geometric average of Ct (threshold cycle) values of the internal reference gene *Actin 11* [34].

## Results and Discussion

### Resistance and susceptibility reaction of genotypes

Female cysts were counted after 30 dpi with SCN as described by Poromarto and Nelson [13]. Average Female indices of 13.5 and 40.3 were obtained for PI 533561 and GTS-900, respectively. According to the Schmitt and Shannon scale [35] soybean reactions to SCN are categorized as the following: FI <10 = resistant, FI 10 to 30 = moderately resistant, FI 31 to 60 = moderately susceptible, and FI >60 = susceptible. The same scale was used to classify different market classes of common bean in previous studies [12, 13]. Therefore, PI533561 was considered moderately resistant and GTS-900 was considered moderately susceptible, which corroborated the earlier studies conducted on bean genotypes [13, 14].

### Ion-Torrent sequencing and aligning to the reference genome

For an in-depth analysis of the transcriptional reprogramming induced in infected common bean roots, RNA-seq was carried out on isolated RNA 8 days after infection of resistant and susceptible pinto bean genotypes. The RNA-seq method generates absolute information rather than relative gene expression measurements; thus, it avoids many of the inherent limitations of microarray analysis. This method was used to analyze differences in gene expression between the resistant and susceptible genotypes. We sequenced two biological replicates of cDNA libraries from the four treatments, RC, RI, SC and SI. The number of sequence reads generated ranged between ~ 3.2 and 5.7 million per library as presented in Table 1. Each library produced reads ranging from 50–250 bp in length. GC percent of sequence data from the four libraries were all approximately 46–49% for each library. Approximately 70–93% of the filtered reads from RC, RI, SC and SI libraries, aligned uniquely to the reference genome and were used for further analysis.

**Table 1:** Statistics of raw data for *P*. *vulgaris* L. transcriptome in resistant (R) and susceptible (S) genotypes 8 days post inoculation (I) and non-inoculation (C).

Line	Replicates	Total Reads	Filtered Reads	Uniquely Aligned Reads	% alignment
RI	1	5052999	2968917	2470450	83.21047709
RI	2	3471704	3471704	2714159	78.17944733
RC	1	5302648	4742298	4378694	92.3327467
RC	2	3295422	3263054	2355402	72.18397244
SI	1	3548737	3161497	2798816	88.52818775
SI	2	4413126	4395535	3216547	73.17759954
SC	1	5694734	5091987	4753670	93.35589427
SC	2	5416888	5395339	3805033	70.52444712

### Differentially expressed genes among the SCN inoculated vs. non-inoculated plants

**Fig 1:**
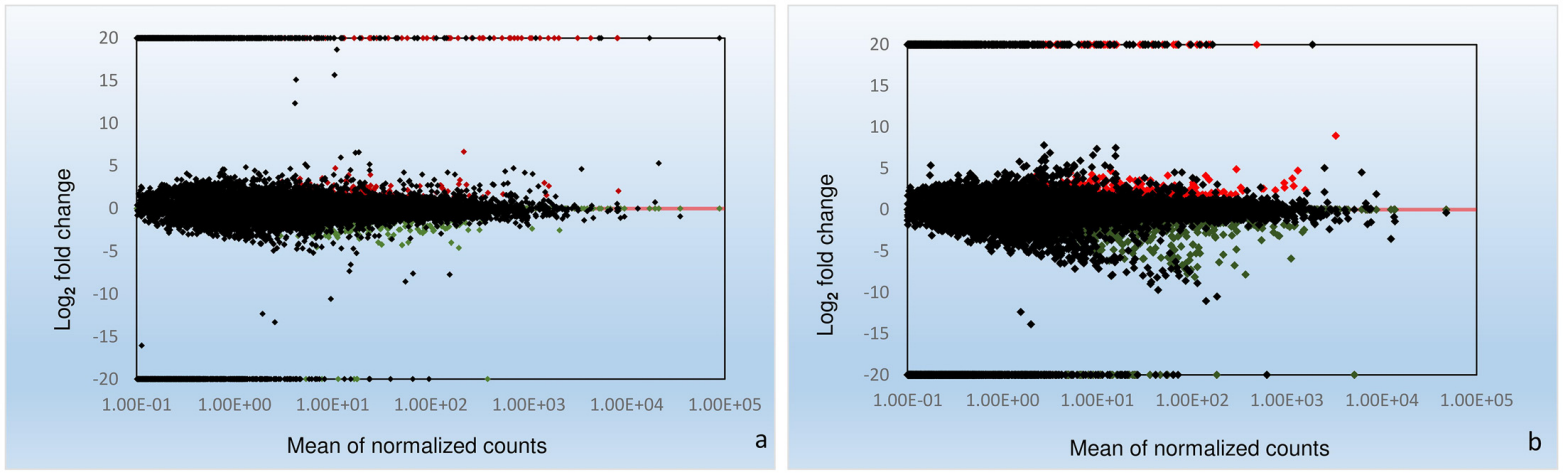
Transcriptome dynamics reveal different patterns of gene regulation in resistant and susceptible common bean–*Heterodera glycines* interactions. MA-plots displaying the log_2_ fold change in the expression plotted against mean of normalized counts in a. resistant genotype inoculated vs. resistant genotype non-inoculated (RI vs. RC) b. susceptible genotype inoculated vs. susceptible genotype non-inoculated (SI vs. SC). Y-axes indicate fold change values (p<0.05) and x-axes indicate mean of normalized counts. Differentially expressed genes (DEGs) are shown in green, black and red indicating down-regulated, no change and up-regulated genes respectively.

**Fig 2:**
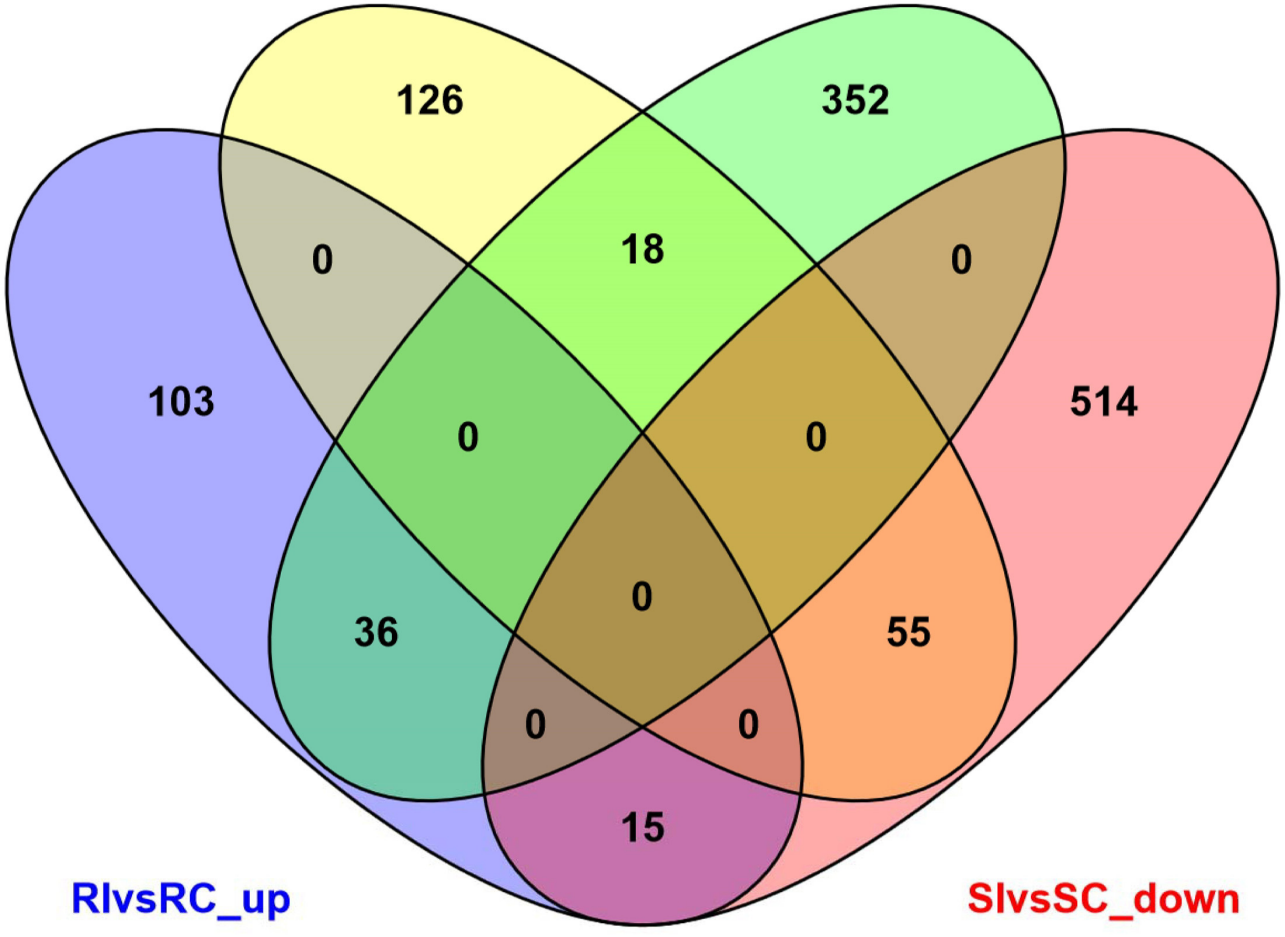
Venn diagram showing differentially expressed genes in common bean–*Heterodera glycines* interactions. RIvsRC up = up-regulated genes in resistant genotype after inoculation (blue); RIvsRC_down = down-regulated genes in resistant genotype after inoculation (yellow); SIvsSC_up = up-regulated genes in susceptible genotype after inoculation (green); SIvsSC_down = down-regulated genes in susceptible genotype after inoculation (red). Common genes between these treatments are shown in the shaded areas. RI and RC = resistant genotypee inoculated and non-inoculated, respectively; SI and SC = susceptible genotype inoculated and non-inoculated, respectively.

**Fig 3:**
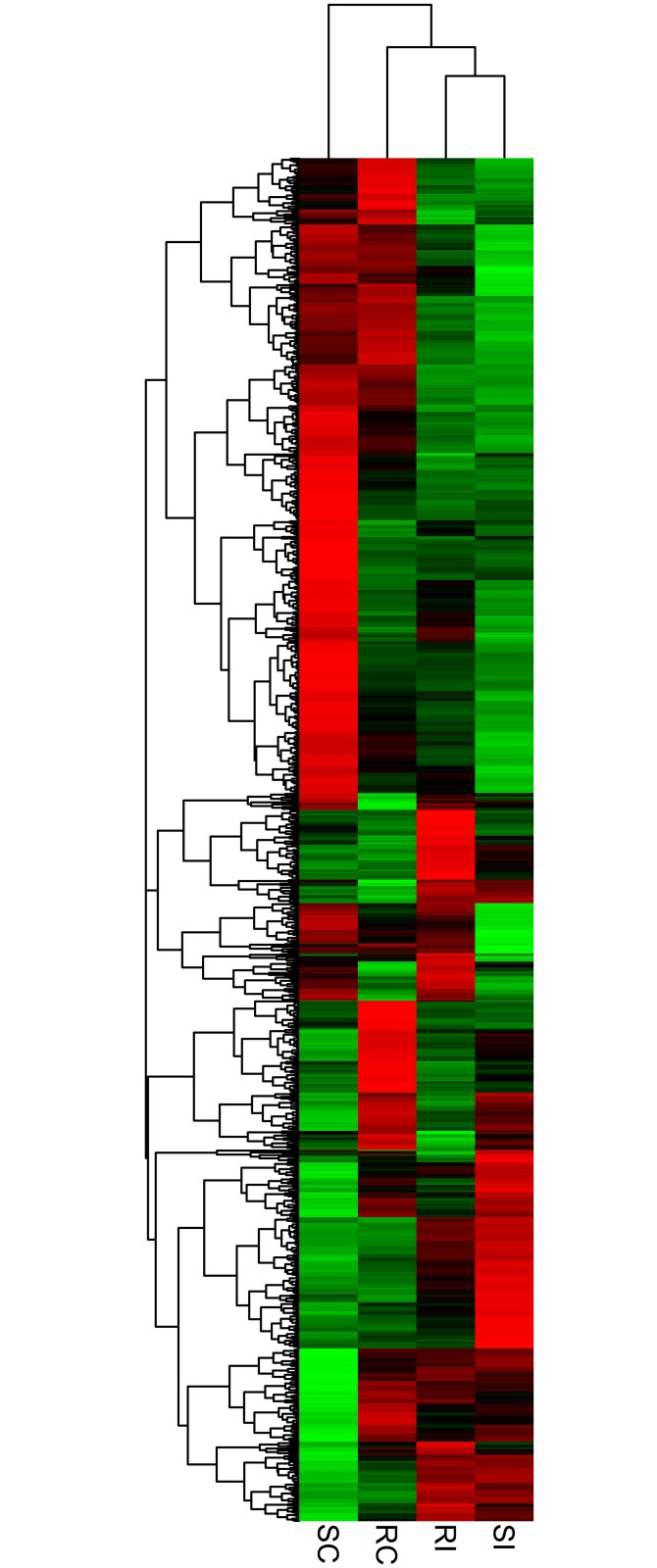
Expression analysis of differentially expressed genes in response to common bean–*Heterodera glycines* interaction. Heat maps are arranged according to their hierarchical clustering based on expression pattern of the genes. From left to right, the four columns respectively show the expression of genes in the non-inoculated susceptible genotype (SC), the non-inoculated resistant genotype (RC), the SCN inoculated resistant genotype (RI) and the inoculated susceptible genotype (SI). Green, black and red indicate low, no change and high expression levels of genes, respectively.

The fold change for transcripts in the four treatments were used as an index for estimating the relative abundance and expression levels between SCN-infected common bean roots and non-infected roots. Transcriptome dynamics of both inoculated genotypes of *P*. *vulgaris* compared to non-inoculated samples were visualized using MA-plots (the log_2_ fold change in expression plotted against mean of normalized counts; (Fig 1)). A large set of genes were differentially expressed in both the genotypes (S1 and S2 Tables). There were 353 DEGs between RI and RC treatments of the resistant genotype PI533561, of which 154 genes were up-regulated and 199 genes were down-regulated. In the susceptible genotype GTS-900, 990 DEGs were detected between SI and SC samples, among which expression levels of 406 genes were up-regulated, and 584 genes, were down-regulated. Despite their different genetic backgrounds, 124 genes that showed differential expression were common between GTS-900 and PI533561 genotypes (Fig 2). The two genotypes shared 36 up-regulated DEGs and 55 down-regulated DEGs, which indicates some common pathways for the response to SCN infection. Fifteen of the up-regulated genes in the resistant genotype were down-regulated in the susceptible genotype following infection. Furthermore, 18 genes showed down-regulation in the resistant genotype that were up-regulated in the susceptible genotype in response to SCN (Fig 2). A heat map of differentially expressed genes (Fig 3) clearly shows both resistant and susceptible genotypes have some similar responses to SCN infection for some DEGs. DEGs were annotated as per the highest amino acid homology with the corresponding annotation and GO categories were assigned to them (S3 and S4 Tables). The data illustrated differences between the resistant and susceptible genotypes during the interaction with SCN and deep analysis of these genes may shed light on the resistance mechanism in common bean. However, some of the changes in gene expression will be due to the difference in genetic background of these genotypes.

Insights into the SCN-soybean interaction were obtained through microarray studies and the identification of genes involved in metabolism, energy, defense and other host pathways that were associated with SCN resistance [36, 37, 38, 39]. RNA-seq has been used in some recent studies involving soybean/SCN interactions [21, 22, 40, 41]. Resistant and susceptible responses of the soybean genotype Peking to incompatible and compatible populations of SCN were studied by Kink et al. [37, 38]. They observed that during the first attempts of the nematode to establish in the host the gene expression patterns in roots were similar in both the susceptible and resistant reactions [37, 38]. Gene expression patterns were different however, during the second phase of infection depending on the resistance/susceptibility of the plant, however syncytia were formed in both cases [38, 42, 43]. At 8 dpi, the syncytia degraded during the resistance response, whereas the syncytia were still developing and provided nourishment to the nematode in the susceptible interaction [37]. In the soybean genotype Peking, differences in gene expression in the syncytium are specific transcriptomic responses during both the resistant and susceptible reaction to two different populations of SCN [42].

**Fig 4:**
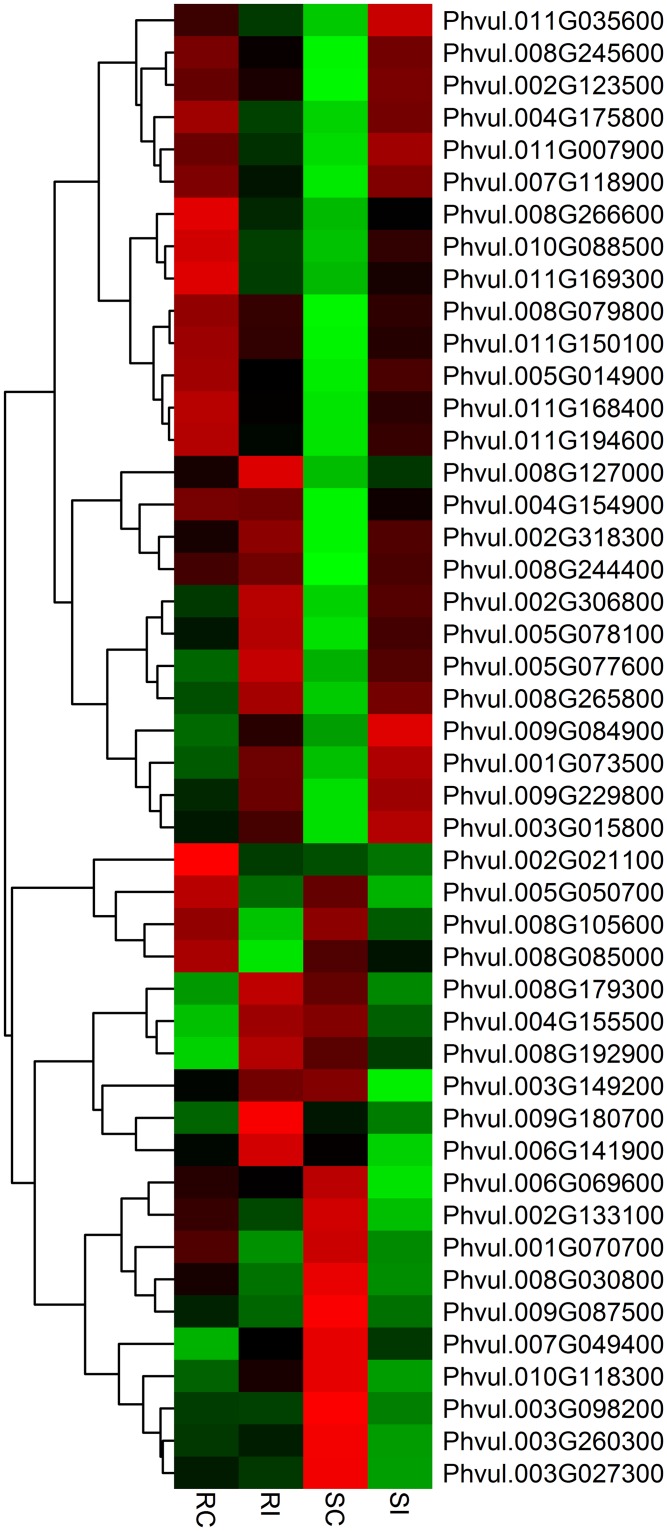
Expression analysis of differentially expressed kinases in response to common bean–*Heterodera glycines* interaction. Heat maps are arranged according to their hierarchical clustering based on expression pattern of the genes. From left to right, the four columns respectively show the expression of genes in the non-inoculated resistant genotype (RC), the SCN inoculated resistant genotype (RI), the non-inoculated susceptible genotype (SC) and the inoculated susceptible genotype (SI). Green, black and red indicate low, no change and high expression levels of genes, respectively.

There are substantial reports regarding the role of kinases in stress response in common bean [44, 45]. Kinase signaling appears to have an important role in the resistance/susceptibility reaction of common bean genotypes in response to pathogens [46, 47]. In soybean, up-regulation of the MAP3K homolog implicated the role of mitogen-activated protein kinase signaling in the regulation of resistance to SCN [39]. A heat map of differentially expressed kinase genes is shown in Fig 4. Different classes of protein kinases were upregulated and downregulated in the resistant genotype. One of the upregulated DEGs (Phvul.009G180700) was annotated as a leucine-rich repeat protein kinase family protein. On the other hand, 27 protein kinases were induced and 18 protein kinases were down-regulated in the susceptible genotype. One of the transmembrane protein kinase tmk1-like genes (Phvul.003G015800) was upregulated in GTC-900 and has 51% similarity with the *Rhg4* like gene (GeneBank ID: NP_001236710) in soybean.

**Fig 5:**
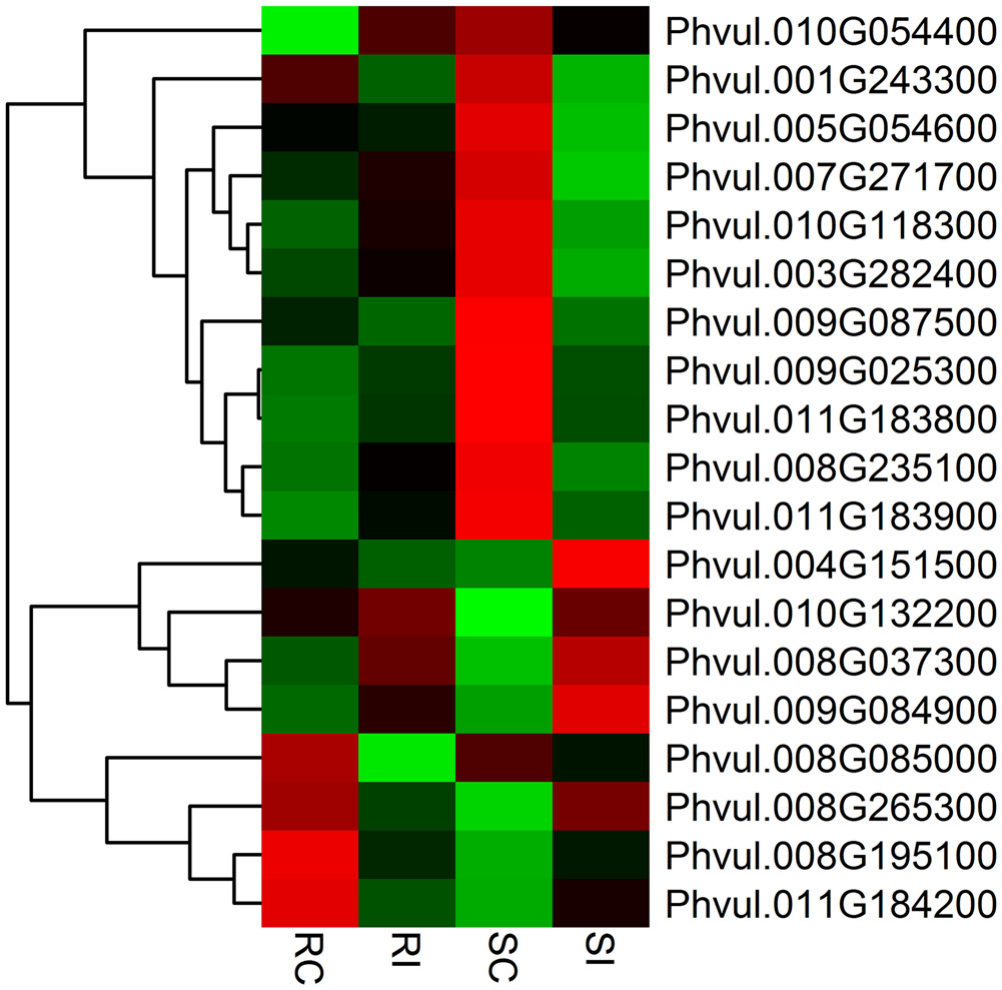
Expression analysis of differentially expressed defense related genes in response to common bean–*Heterodera glycines* interaction. Heat maps are arranged according to their hierarchical clustering based on expression pattern of the genes. From left to right, the four columns respectively show the expression of genes in the non-inoculated resistant genotype (RC), the SCN inoculated resistant genotype (RI), the non-inoculated susceptible genotype (SC) and the inoculated susceptible genotype (SI). Green, black and red indicate low, no change and high expression levels of genes, respectively.

**Fig 6:**
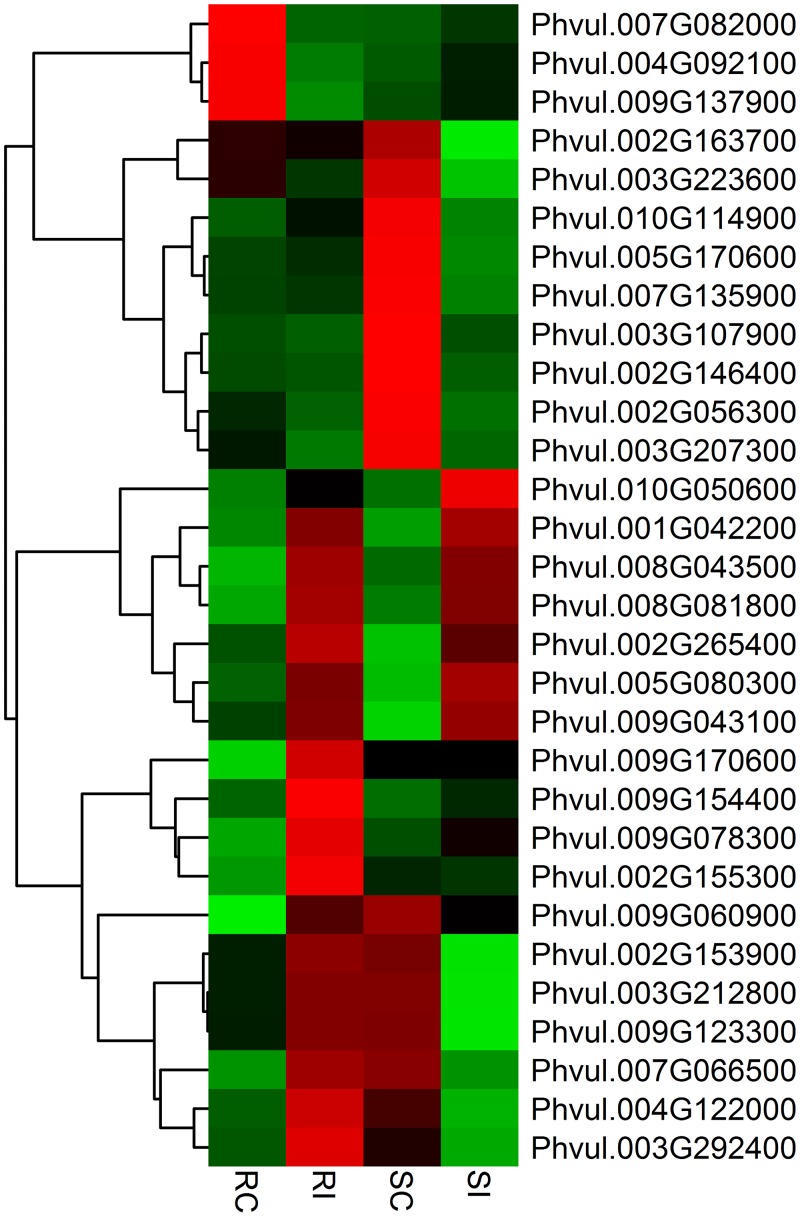
Expression analysis of differentially expressed transcription factors in response to common bean–*Heterodera glycines* interaction. Heat maps are arranged according to their hierarchical clustering based on expression pattern of the genes. From left to right, the four columns respectively show the expression of genes in the non-inoculated resistant genotype (RC), the inoculated resistant genotype (RI), the non- inoculated susceptible genotype (SC) and the inoculated susceptible genotype (SI). Green, black and red indicate low, no change and high expression levels of genes, respectively.

Heat maps of defense related genes and transcription factors (TFs) are presented in Figs 5 and 6, respectively. Nineteen DEGs were found to be associated with defense related functions and two of them (Phvul.010G132200 and Phvul.008G037300) were upregulated in both genotypes. An important candidate gene for SCN resistance may be the disease resistance protein TIR-NBS-LRR (Phvul.010G054400), which was expressed only during SCN infection in the resistant genotype. The most represented group of disease resistance genes (R genes) in plants cloned to date are NLR proteins characterized by nucleotide-binding site (NBS) and leucine-rich repeat (LRR) domains as well as variable amino- and carboxy-terminal domains [48]. These large proteins are abundant in plant genomes and are actively involved in plant resistance to diverse pathogens such as bacteria, viruses, fungi, nematodes, insects and oomycetes [49]. For example, two homologues of a leucine-zipper, nucleotide-binding site, leucine-rich repeat (LZ-NBS-LRR) R gene family, *Gpa2 and Rx1* genes, confer resistance to potato virus X, and potato cyst nematode (*Globodera pallida*) respectively [50]. To support the functional correlation between NLR genes and disease resistance in soybean, genome wide QTL analysis was conducted and the analysis determined that QTLs located near the NLR gene cluster were not specific for one particular disease but rather for several pathogens and pests including fungi, bacteria and nematodes [51]. Most importantly, NLR resistance proteins were involved in regulating SCN interactions in SCN resistant soybean varieties when examined with whole-genome gene expression profiling [41]. Further molecular characterization of NLR genes in SCN resistant common bean will help elucidate the resistance mechanism to SCN and other pathogens.

Our analysis revealed that expression of 30 TFs changed significantly in response to SCN infection in both common bean genotypes. Two WRKY transcription factors (Phvul.001G042200 and Phvul.008G081800) were upregulated in the resistant plants after SCN infection while 5 WRKY transcription factors (Phvul.001G042200, Phvul.002G265400, Phvul.005G080300, Phvul.008G081800, Phvul.009G043100) were upregulated in the susceptible plants. WRKY DNA-binding protein 40 (Phvul.001G042200) and WRKY DNA-binding protein 70 (Phvul.008G081800) were upregulated in both the genotypes indicating similar response of those factors to SCN infection. The role of WRKY transcription factors was investigated in regulation of reactive oxygen species (ROS) through genetic analysis with double mutants in *Arabidopsis* by Brosché et al. [52]. The results indicated that these TFs were involved in regulation of ROS induced programmed cell death along with SGT1b, a component of ubiquitin mediated protein degradation. A key role of ROS was found in the hypersensitive response during R-mediated resistance mechanisms to different pathogens [53] and genes involved in regulating ROS are often up-regulated [52]. Genes having a role in ROS scavenging pathways such as glutathione transferase, heat shock proteins and peroxidases were differentially expressed in both the genotypes. Differential expression of genes related to cell wall synthesis and remodeling in common bean were also shown in this study. The components of plant cell walls are modified during attack by pathogens as their first line of defense [54] and cell wall degradations occur during syncytium development due to the cell wall modifying and degrading proteins produced in cyst nematode infections [55]. The structural cell wall proteins such as hydroxyproline-rich glycoprotein (HRGPs) are catalyzed by an oxidizing system based on peroxidase and H_2_O_2_ and contribute to the strength of cell walls during pine wood nematode responses [56]. Two HRGPs (Phvul.001G044200 and Phvul.005G135700) were upregulated during the resistance response to SCN infection in the present study. In soybean, several genes involved in cell wall remodeling were differentially expressed in roots after infection by SCN [38, 57]. Similar responses were also observed in soybean after root knot nematode (*Meloidogyne incognita*) infection [58]. The balance between the formation of ROS and changes in the transcripts for cell wall remodeling enzymes seems to be important for the reaction of plants under stress conditions [59]. Interestingly, photorespiration is considered an important part of stress response in plants for preventing ROS accumulation and plays a major role in the readjustment of redox homeostasis [60]. In this study, 24 photosynthesis related genes were down regulated in the resistant genotype, while 38 photosynthesis related genes were down regulated in the susceptible genotype. This indicates suppression of the photosystem in the resistant and susceptible genotypes after SCN infection. A similar response was observed with SCN infection in soybean plants where both photosystem I and II were suppressed [22]. Different studies suggest that downregulation of photosystem activity is an adaptive response to biotic attack due to transition from growth- to defense-oriented metabolism [61].

The differential expression of pathogenesis-related (PR) genes under various abiotic and biotic stresses is a well-known phenomenon in legumes [62, 63]. Expressions of pathogenesis related genes PR-1, PR-2 and PR-5 were highly induced in *Arabidopsis* roots, but expression of PR-4 was not altered during infection by root-knot nematode [64]. Overexpression of these genes in roots may be explained by the potential functions of PR genes such as in cell wall modification and osmotic regulation [62]. In the current study, one PR-5 like receptor kinase (Phvul.004G155500) was up-regulated in the resistant genotype while one pathogenesis related family protein (Phvul.005G081500) was up-regulated in the susceptible genotype. One CAP (Cysteine-rich secretory proteins, Antigen 5, and Pathogenesis-related 1 protein) superfamily protein (Phvul.006G197100) was found down-regulated in resistant genotype after SCN infection. Similar kinds of responses were observed in soybean after SCN infection including a highly upregulated CC-NB-LRR gene, several heat shock protein genes and heat shock TFs, defense related WRKY TFs and PR genes [22, 39]. Molinari et al. [65] demonstrated that several PR proteins are differentially expressed after root-knot nematode infection in tomato. Nematode infection elicited salicylic acid-dependent systemic acquired resistance in plants. Similar pathways may be involved in dry bean in response to SCN infection, however further research is needed to confirm this hypothesis.

### Gene ontology analysis of differentially expressed genes

**Fig 7:**
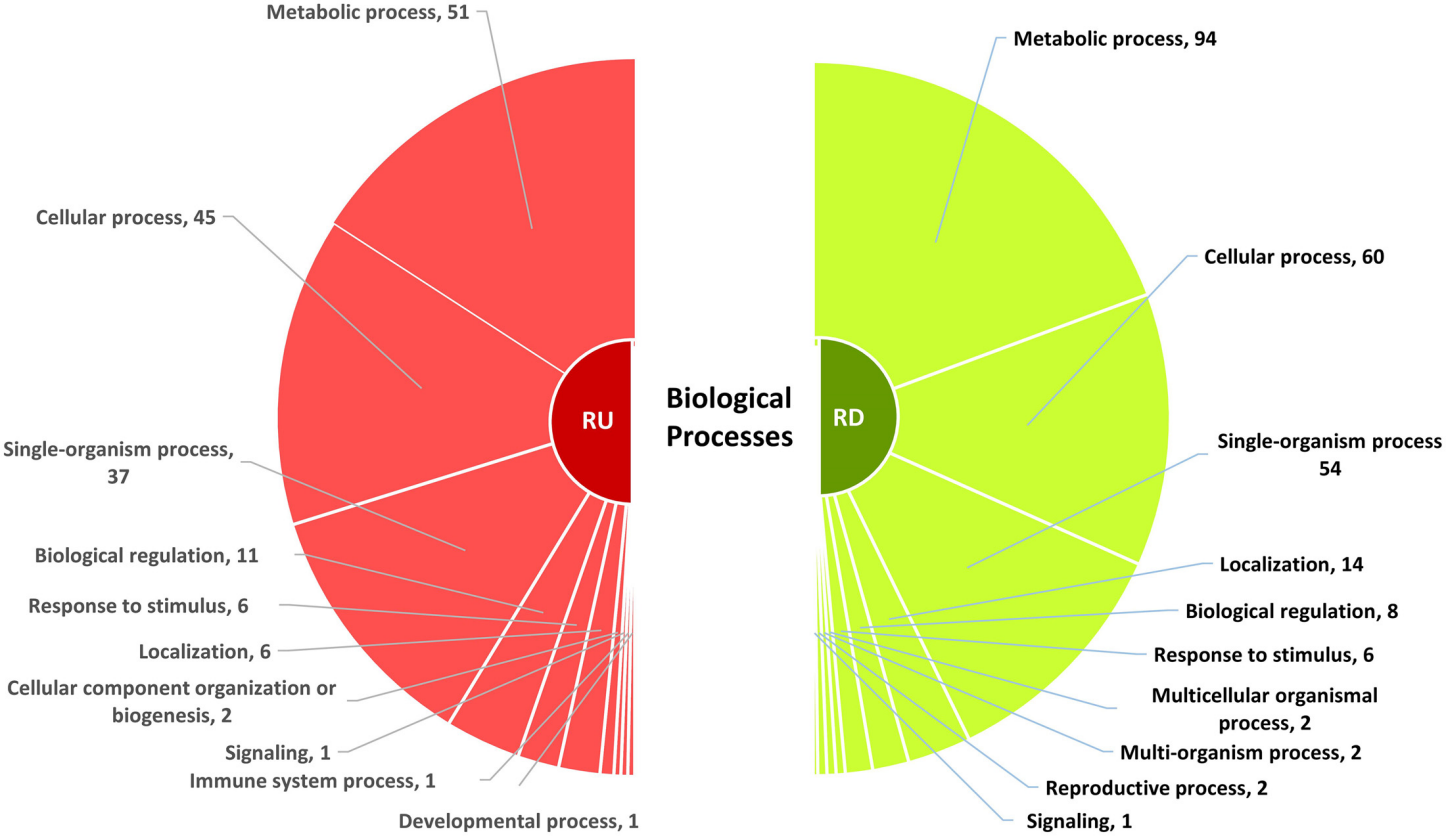
Classification of differentially expressed genes based on biological processes in a common bean genotype resistant to *Heterodera glycines*. Significant gene ontology categories (p < 0.05) along with the number of genes involved are indicated for differentially expressed genes. RU = resistant upregulated; RD = resistant downregulated.

**Fig 8:**
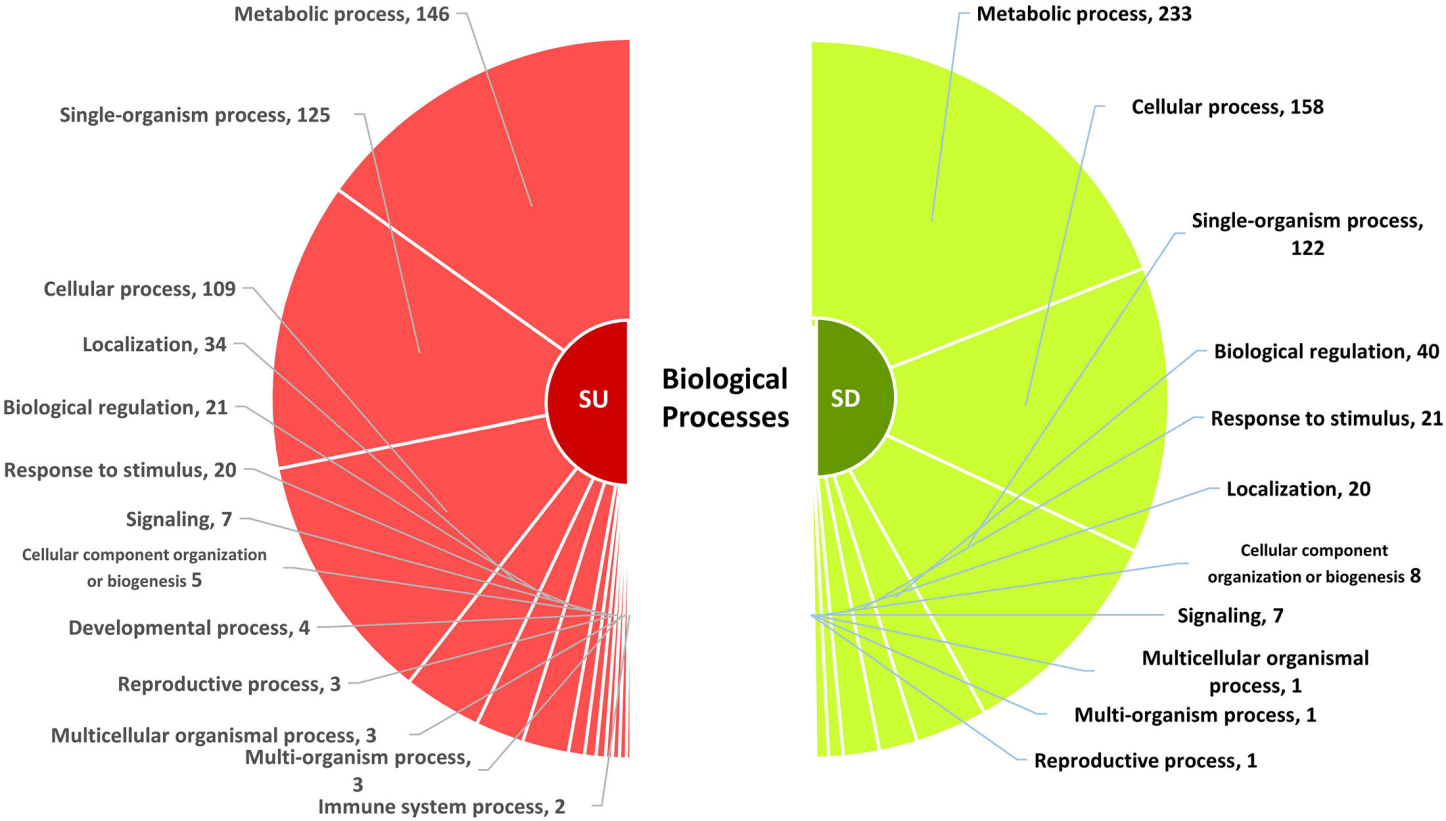
Classification of differentially expressed genes based on biological processes in a common bean genotype susceptible to *Heterodera glycines*. Significant gene ontology categories (p < 0.05) along with number of genes involved are indicated for differentially expressed genes. SU = susceptible upregulated; SD = susceptible downregulated.

Differentially expressed genes in resistant and susceptible genotypes according to significant gene ontology (GO) under ‘biological processes’ categories are presented in Figs 7 and 8, respectively (S5 and S6 Tables). A major reprogramming of plant metabolism appears to occur during nematode-plant interactions. DEGs were represented into 10 different categories based on biological process through gene ontology (GO) in the resistant genotype. A significant number of genes (51 upregulated and 94 downregulated) were categorized under the GO term ‘GO:0008152’ representing metabolic process. Other apparent GO terms associated with differentially expressed genes were cellular process (GO:0009987) and cellular metabolic process (GO:0044237). Several GO terms, for example, single-organism metabolic process, transport, primary metabolic process, stress or defense responses, biotic stimulus, abiotic stimulus, signaling responses and cellular signaling were enriched in the resistant genotype. In the susceptible genotype, the enriched GO terms included cellular process (GO:0009987), metabolic process (GO:0008152), single organism process (GO:0044699), biological regulation (GO:0065007) and response to stimulus (GO:0050896). Nitrogen compound metabolic process (GO:0006807) seems to play an important role as this was enriched in both genotypes. Interestingly, a decrease in photosynthetic activity (GO:0015979) due to SCN infection was also observed in both genotypes. Hosseini and Matthews [40] also demonstrated that photosynthesis was one of the most suppressed GO processes in soybean following SCN infection. Suppression of photosynthesis related genes have also been observed in other pathogen-plant interactions as well. For example, transcripts encoding for ribulose bisphosphate carboxylase and chlorophyll a/b-binding proteins were down-regulated during fungal infection in common bean [66].

### Validation of some differentially expressed genes

**Fig 9:**
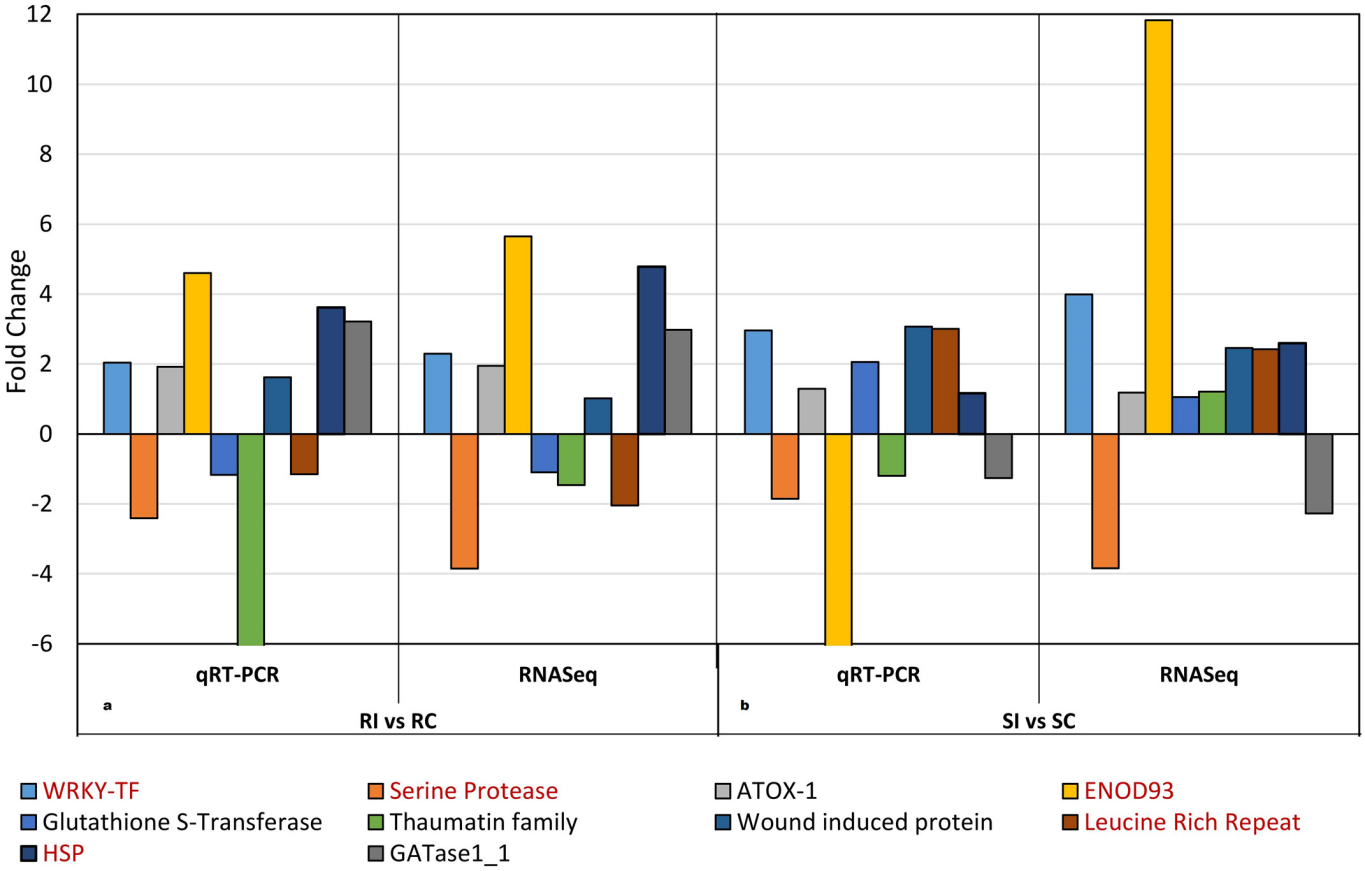
Quantitative RT-PCR (qPCR) validation of the relative expression levels of genes selected from the RNA-seq analysis of common bean–*Heterodera glycines* interaction. Expression profiles of 10 genes (color coded) as determined by qPCR and RNA-seq. The signal intensity of each transcript was normalized using Actin11. The x-axis indicates two groups of comparisons: a. resistant genotype inoculated vs. resistant genotype non-inoculated (RI vs. RC). b. susceptible genotype inoculated vs. susceptible genotype non-inoculated (SI vs SC). The y-axis shows the fold change increase/decrease in expression level of the genes.

In order to validate the RNA-seq data, ten DEG having annotations in the reference genome were selected for quantitative real time PCR (qPCR) analysis. Locus number, functional annotation and primer sequences of these genes are presented in Table 2. These were genes encoding WRKY transcription factor, serine protease, ATOX1, glutathione S-transferase, wound induced protein, leucine rich repeat, heat shock protein, ENOD93, Thaumatin family and GAT ase1_1. Relative expression profiles of DEGs in the resistant genotype evaluated using qPCR were in complete agreement with the RNA-seq data as depicted in Fig 9a. Similar expression patterns were observed in the susceptible genotype when qPCR and RNA-seq data were compared except for two genes (ENOD93 and Thaumatin family) as shown in Fig 9b. RNA for qPCR was extracted from different biological replicates than RNaseq samples therefore variability may exist in treatment/conditions. The WRKY transcription factor (Phvul.002G265400) (Fig 6) was also upregulated in both resistant and susceptible genotypes after SCN infection as occurred in their corresponding non-inoculated controls. WRKY transcription factors are known to take part in defense responses to viral, bacterial, fungal and nematode pathogens [39, 67, 68], which correlates with the present study. Likewise, Genes encoding wound induced protein (Phvul.006G102300) and heat shock protein (Phvul.011G016100) showed higher expression in both genotypes in RNA-seq and qPCR experiments. This agrees with the other studies on the involvement of these genes in abiotic and biotic stress responses [39, 41, 68]. Disease resistance proteins with leucine rich repeats, protein kinases, zinc finger domain proteins, RING domain proteins, cytochrome P450s, MYB and WRKY transcription activation families were identified in a genome wide association study conducted on soybean in search of genetic factors involved in SCN resistance [69]. These genes possibly play an important role in mediating plant pathogen interactions, and therefore may be potential targets to manipulate for enhancing common bean resistance to SCN.

**Table 2:** List of primers to amplify the selected genes for qPCR analysis.

Primer name	Gene	Functional annotation	Forward Primer (5'-3')	Reverse Primer (5'-3')
WRKY-TF	Phvul.002G265400	WRKY transcription factor	AAGTCCGTGAAGAGCAATCC	TGTCCCTTTCCACCCTTTTC
Serine Protease	Phvul.001G238100	Subtilisin/Kexin-Related Serine Protease	ATGAAGGATGAGGATTCTGCG	GCAAAGCCACTGAAACTTCG
ATOX-1	Phvul.004G107100	Copper Transport Protein ATOX1-Related	ACAGCATCTGGCCTTTCTG	GTGACACCATTTTCGTAGCTTTG
ENOD93	Phvul.009G198400	Early nodulin 93 ENOD93 protein	GGCAAGAGCCAATCTTAATCAC	TGAGAATGTGAGGGTGGAATG
Glutathione S-Transferase	Phvul.008G113600	Glutathione S-Transferase	CGGAGTCCCTTGTGATTGTT	ACTGATTTCCACGTAGCACC
Thaumatin family	Phvul.008G118200	Thaumatin family	GAGCTCAAACAATGGCAAGAG	GCACCTCCAAGTATCCCTG
Wound induced protein	Phvul.006G102300	Wound-Induced Protein WIN-Related	GCTTTCTGTGGACCCTCTG	CTAATATCCAAGTCCAAGCCCC
Leucine Rich Repeat	Phvul.011G169300	Leucine Rich Repeat	CTTGACTATGAGCTTGTCCCC	TGCTTTCTCTGTAAGGTGTCC
HSP	Phvul.011G016100	Molecular chaperone (small heat shock protein)	CTTTCAACACCAACGCCATG	GCTCAAGCTCCGAGTAGG
GATase1_1	Phvul.002G214100	GATase1_1; Subgroup of proteins having the Type 1 glutamine amidotransferase	TCGTGACCTTCTCAACTTGC	ACCAATGTCCCAACCAGTAG

## Conclusion

This is the first study on molecular responses of common bean during SCN infection. RNA sequencing technology was used to identify differentially expressed genes 8 days after inoculation of SCN resistant and susceptible genotypes of common bean. Importantly, we identified multiple TFs and protein kinases that were modulated by SCN infection. The differentially expressed genes identified are reported to be involved in similar pathways in the closely related host-pathogen interaction of soybean-SCN. Functional classification based on GO and enrichment analyses demonstrated up-regulation of genes for 10 different categories in biological processes such as metabolic process, cellular process, single-organism process and response to stimuli. RNA-seq data was further validated through qPCR. Further characterization of some of the differentially expressed genes may lead to genetic engineering by over-expressing defense related genes or silencing genes that promote syncytium formation to manage the plant parasitic nematode infestations. Overall, comparative transcription profiling provided valuable information on SCN resistance in common bean, which can aid future development of resistant common bean varieties.

Research extended our understanding of the genetic architecture of SCN resistance in two different genetic backgrounds. The results also have potential implications for comparative gene discovery in legumes and development of new resources for identifying markers for marker assisted selection. Functional characterization of some of the candidate genes and genetic mapping of SCN resistance using the same genotypes used in this study are currently underway.

## Supporting Information

S1 Table List of genes differentially expressed in resistant genotype after SCN infection. (XLSX)

S2 Table List of genes differentially expressed in susceptible genotype after SCN infection. (XLSX)

S3 Table Functional annotation of differentially expressed genes in resistant genotype after SCN infection. (XLSX)

S4 Table Functional annotation of differentially expressed genes in susceptible genotype after SCN infection. (XLSX)

S5 Table GO and enrichment analysis of differentially expressed genes in resistant genotype after SCN infection. (XLSX)

S6 Table GO and enrichment analysis of differentially expressed genes in susceptible genotype after SCN infection. (XLSX)
